# Considerations regarding current diagnosis and prognosis of hepatocellular carcinoma

**Published:** 2015

**Authors:** CG Cristea, IA Gheonea, LD Săndulescu, DI Gheonea, T Ciurea, MR Purcarea

**Affiliations:** * Department of Gastroenterology, Craiova University of Medicine and Pharmacy, Romania; ** Department of Radiology, Craiova University of Medicine and Pharmacy, Romania; *** Department of Urology, Nephrology, Dermatology, Transplant Immunology, “Carol Davila” University of Medicine and Pharmacy of Bucharest, Romania

**Keywords:** hepatocellular carcinoma, alpha-fetoprotein, contrast-enhanced ultrasonography, multi-detector CT, diffusion weighted MRI

## Abstract

Hepatocellular carcinoma is a major health issue, ranked the fifth most common tumor and currently being responsible for a third of the cancer-related deaths globally, with an ever-increasing number of fatalities. Current advances in contrast-enhanced imaging techniques such as contrast-enhanced ultrasonography, multi-detector computed tomography and diffusion-weighted magnetic resonance imaging are improving the rate of hepatocellular carcinoma diagnosis. Contrast-enhanced ultrasonography has widely become the first choice in liver tumor assessment, as it is faster, simpler and safer than other forms of diagnostic imaging. On the other hand, cross sectional computed tomography is frequently employed when a hepatic formation is suspected of malignancy and allows a more accurate characterization of lesions through multiphasic multi-detector computed tomography technology. Diffusion weighted magnetic resonance imaging represents another addition to the wide range of diagnostic and prognostic techniques available for patients with hepatocellular carcinoma and is currently regarded as one of the best tools for the characterization of these lesions. Furthermore, groundbreaking biomarkers for hepatocellular carcinoma are being discovered, although alpha-fetoprotein remains one of the most frequently used serum test in the early stages. Nonetheless, further advances are required for the detection of small liver carcinomas.

**Abbreviations** : AASLD = American Association for the Study of Liver Diseases, AFP = Alpha-fetoprotein, AFP-L3 = Alpha-fetoprotein isoform 3, CEUS = Contrast-enhanced ultrasonography, DCP = Des-gamma-carboxy-prothrombin, DW-MRI = Diffusion weighted magnetic resonance imaging, FNA = Fine neddle aspiration, Gd-EOB-DTPA = Gadolinium ethoxybenzyl diethylenetriaminepentaacetic acid, GGT-II = Gamma-glutamyl transpeptidase II, GP73 = Golgi protein 73, HCC = Hepatocellular carcinoma, HCCR-1 = Human cervical cancer proto-oncogene 1, IL-18 = Interleukin 18, MDCT = Multi-detector computed tomography, PET-CT = Positron emission tomography – computed tomography, SUV = Standardized Uptake Value

## Introduction

Hepatocellular carcinoma (HCC) is a major health issue, as recognized by the World Health Organization (WHO), being ranked the fifth most common tumor, currently responsible for a third of cancer-related deaths on a global scale, with an ever-increasing number of fatalities. As a result, there have been numerous attempts to establish a set of guidelines for an early liver cancer diagnosis and long-term prognosis based on different serum biomarkers such as alpha-fetoprotein (AFP), des-gamma-carboxy-prothrombin (DCP) or other novel markers that are currently being studied [**1**]. Alternatively, advances in various imaging techniques (contrast-enhanced ultrasonography (CEUS), multi-detector computer tomography (MDCT), diffusion weighted magnetic resonance imaging (DW-MRI) have significantly improved the detection of hepatocellular carcinoma, with limitations of less than 2 cm in diameter in the case of liver tumors, where further studies are required [**2**]. An accurate screening method or a combination of methods for early HCC detection are often evaluated based on sensitivity and specificity rates, as these statistical indicators are not dependent on disease prevalence and offer the possibility of comparing studies performed in populations with varying characteristics [**3**].

In this approach, we describe novel imaging techniques, reviewing their diagnostic and prognostic yield and their applicability to clinical practice, as well as current and future perspectives on biomarkers regarding the evaluation of malignant hepatoma.

**Imaging Techniques**

**Contrast-Enhanced Ultrasonography (CEUS)**

**Fig. 1:**
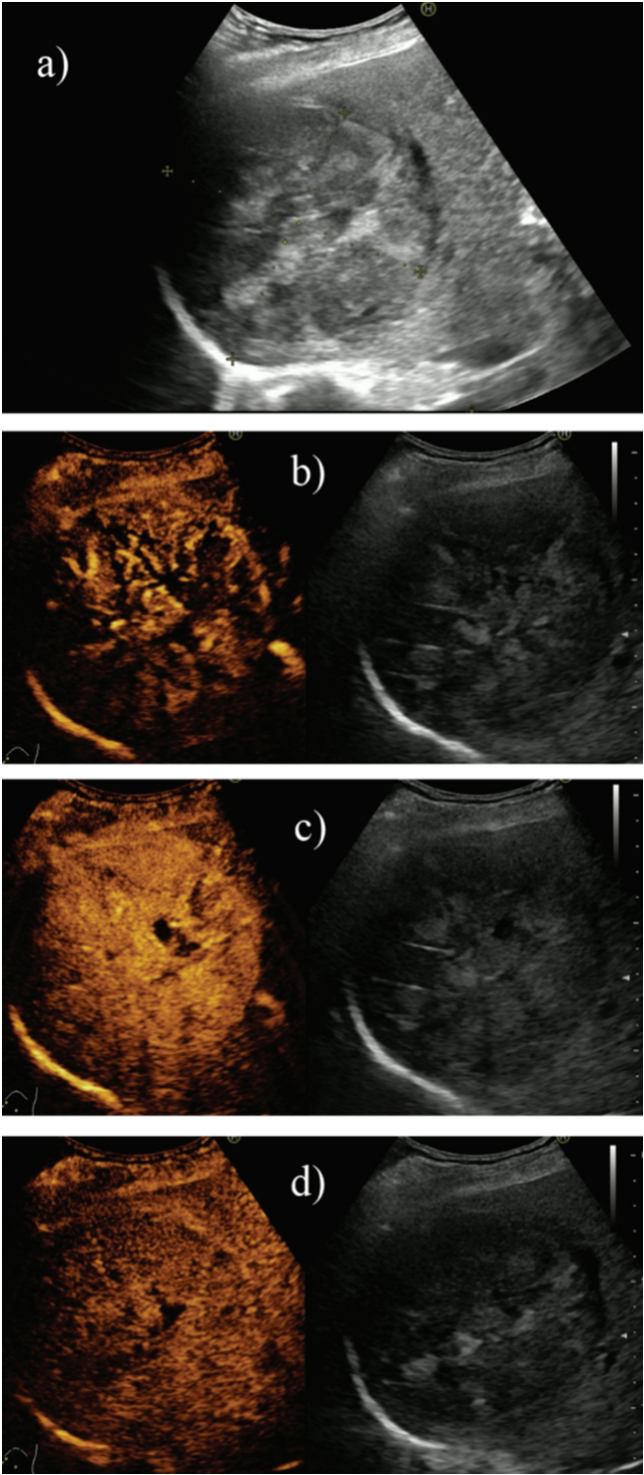
Large HCC (a), which presents on CEUS chaotic intratumoral vascularization (b), incomplete contrast filling during the arterial phase due to areas of tumor necrosis (c), as well as slow and incomplete washout after approximately 5 minutes of examination (d)

**Fig. 2:**
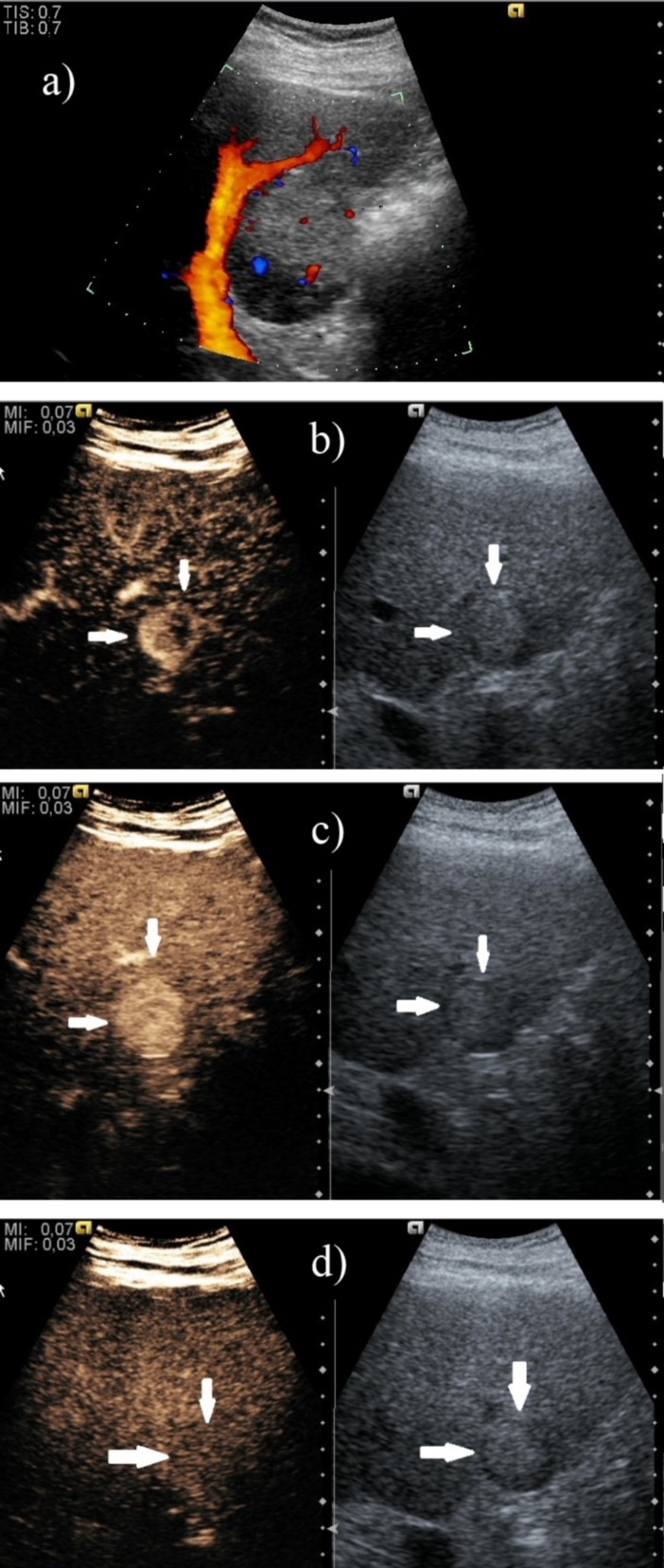
Well-differentiated hepatocellular carcinoma with discreet vascularization on Doppler ultrasound (a), but with intense and heterogeneous hyperenhancement during the arterial phase (b, c) and without washout during the late phase (d)

Contrast-enhanced ultrasonography, a non-invasive diagnostic method for various types of hepatic tumors such as hemangioma, focal nodular hyperplasia or adenoma, has recently been introduced as an imaging technique useful for differentiating HCC from cirrhosis nodules based on the different vascularization patterns of the two pathologic entities [**4**]. The echographic contrast agent approved for use in Europe (SonoVue) is composed of small micro-bubbles, which can flow inside capillaries and, as a result, can generate a map of the intratumoral vascularization with a high degree of accuracy [**5**]. The evaluation of hepatic nodular lesions undergoes three vascular phases: the arterial and portal phases, which occur during the first 2 minutes after the contrast agent is injected, and a late phase that lasts until the contrast is eliminated. The typical appearance of HCC in contrast enhanced ultrasound is usually hyperenhancement in the arterial phase, with a chaotic vascular pattern (**Fig. 1**). In the portal venous and late phases, HCC usually shows hypoenhancement apart from well-differentiated HCC that may be iso-enhancing (**Fig. 2**) [**6**]

**Fig. 3:**
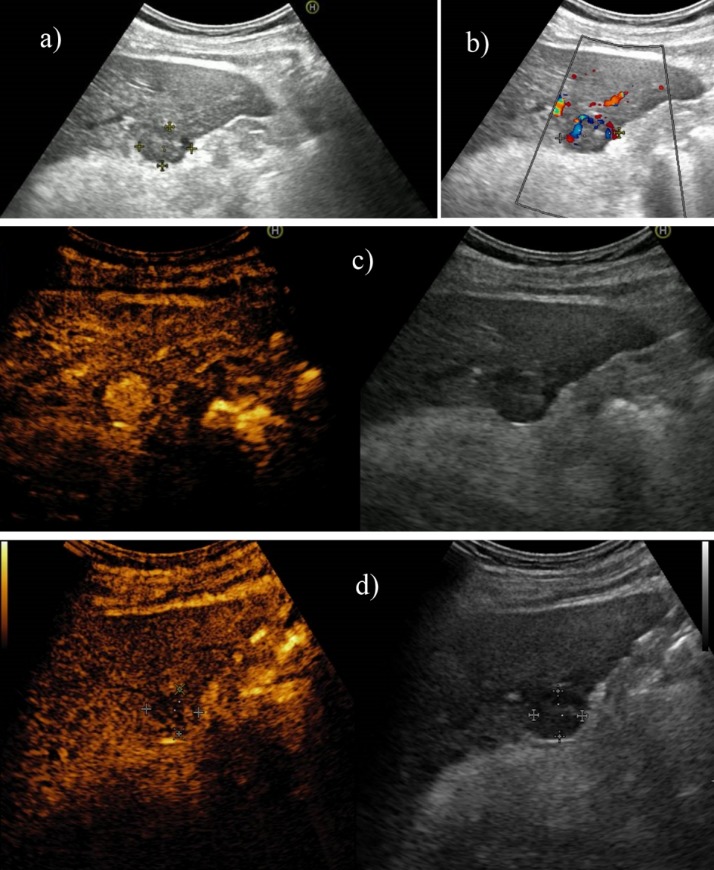
In a 56-year-old patient, standard ultrasound showed a small nodule of 1,8 cm in the hepatic segment V (a) With “basket pattern” type vascularization on Doppler ultrasound (b) In the arterial phase, the nodule fills with contrast faster and more intense compared to the hepatic parenchyma (c) and presents washout in the late phase (d)

Hepatic carcinogenesis is a multistage process, which frequently has a cirrhosis regenerative nodule as a starting point. During CEUS, this type of lesion does not differ from the surrounding parenchyma and, consequently, is not visible in any vascular phase. For this reason, any change in behavior during the arterial phase, such as hypervascular images, can be suggestive for a dysplastic nodule, which can be very difficult to differentiate from early HCC (**Fig. 3**). Although in this type of situation biopsy followed by histopathological analysis is required, most HCCs have a typical behavior in CEUS. Due to their predominant arterial vascularization, during the arterial phase, the malignant nodules can be differentiated from the normal parenchyma and furthermore their nutrition artery may become visible [**5**]. 

Along with being a valuable early diagnosis instrument, CEUS may also provide information regarding the degree of cellular differentiation of the tumor and possible complications such as portal vein thrombosis [**7**], which are relevant for short and long-term prognosis of HCC. A recent study suggests that CEUS can be used to determine whether a HCC is either well or moderately to poorly differentiated based on variances in intra-tumoral vascularization. Thus, in contrast to well differentiated tumors, which present a “fast-in, slow-out pattern”, as tumor grade decreases, the contrast agent tends to washout more rapidly, determining a “fast-in, fast-out pattern”, the result of the increased density of arteriovenous fistulae [**8**].

In spite of certain limitations concerning the reduced accessibility to subdiaphragmatic hepatic segments or to lesions more than 10 cm in depth and the inconvenience of relying on patients to control their breathing [**8**], this method has already proven its diagnostic and prognostic capacity. Regarding this topic, a comparative multicentric study conducted on 267 patients extracted out of 1349, with suspected hepatic lesions after standard ultrasound, revealed that CEUS is comparable to CT in terms of tumor characterization with the added advantage of avoiding radiation exposure [**9**]. Another multicentric prospective study published recently has shown that CEUS has an accuracy of approximately 80% in HCC diagnosis [**10**]. Nevertheless, there is a growing interest in combining ultrasonography with pre-recorded CT or MRI data, an approach with an increased potential to provide even more accurate diagnostic data, as well as to facilitate interventional therapies such as radiofrequency ablation [**11**].

**Computed Tomography (CT)**

**Fig. 4:**
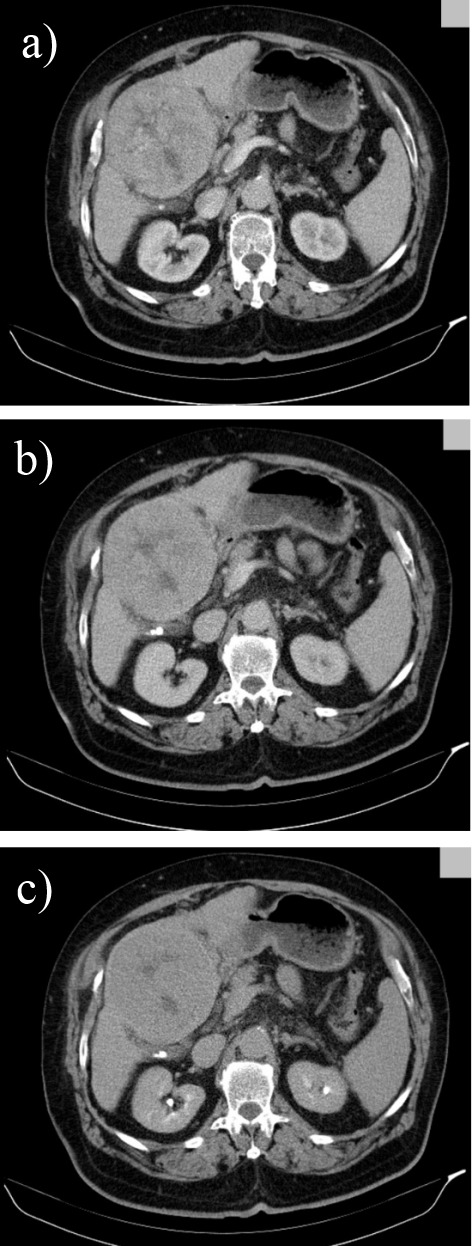
Arterial (a), portal (b) and late phase (c) 16 slices CT in a cirrhotic patient revealing a well-differentiated hepatocellular carcinoma in segment IV with extracapsular extension, heterogenous contrast uptake and central necrosis

Cross sectional computer tomography is frequently the first diagnostic imaging technique employed when a malignant hepatic lesion is suspected and, currently, a more accurate characterization of this type of lesions can be obtained by using multiphasic multi-detector CT technology (**Fig. 4**). This method allows the acquisition of multiple cross sections simultaneously and at rapid speeds, which in combination with an intravenous contrast agent allows a multiphasic analysis of liver parenchyma: a pre-contrast phase, an arterial phase and a portal phase of approximately 30 seconds each and a delayed phase up to 3 minutes [**12**].

**Fig. 5:**
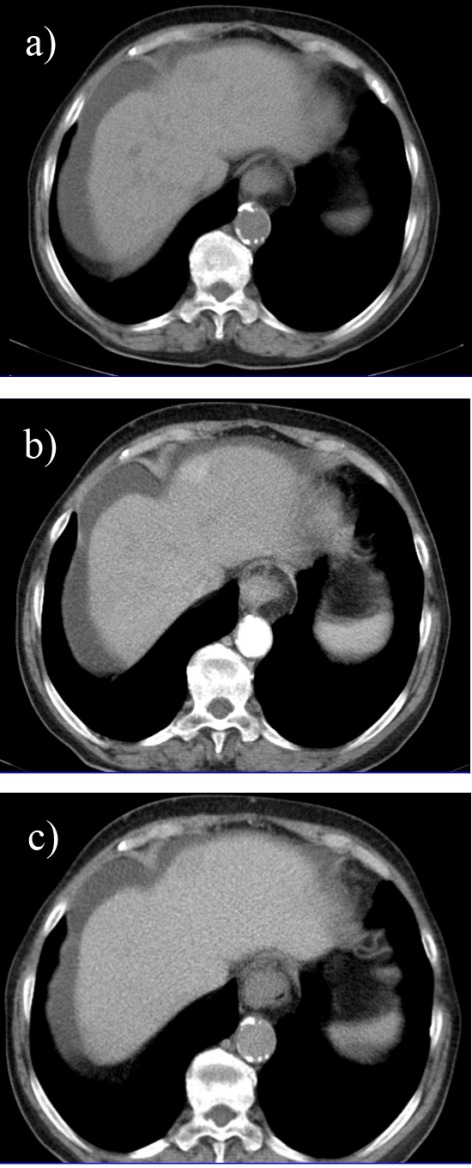
Triphasic multislice CT in a cirrhotic patient showing a moderatly differented small, subcapsular HCC in segment IV with slight hypoattenuation in the precontrast phase (a), with arterial enhancement (b) and washout in the portal phase (c);

**Fig. 6:**
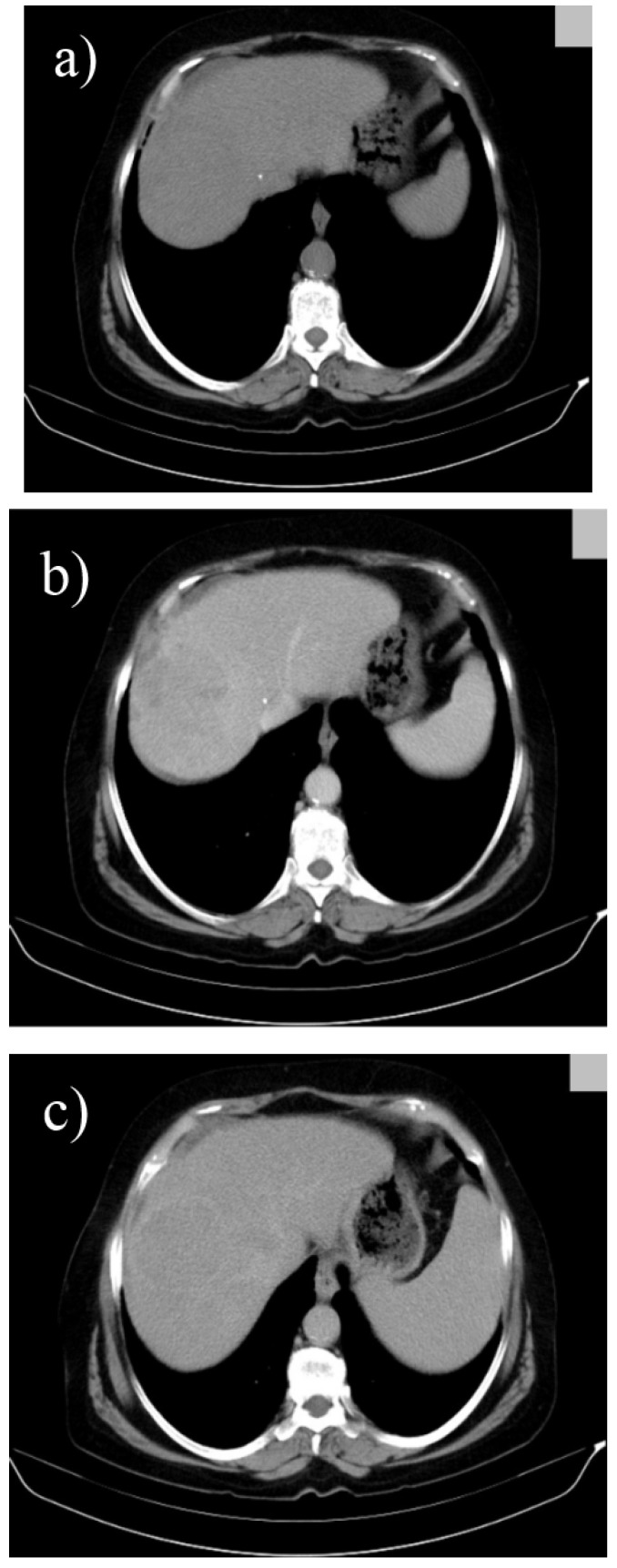
Triphasic multislice CT in a cirrhotic patient showing a well differented HCC in the right lobe with the same density as liver parenchyma in precontrast phase (a), with discrete low density during portal phase (b) and low density with hiperattenuating capsule in the late phase (c)

A 2011 retrospective study conducted over the course of 7 years on 243 patients with histologically confirmed HCC analyzed contrast enhancement patterns on MDCT and their correlation with tumor differentiation based on attenuation, washout and various other case specific factors such as necrosis, aneurysms or dysmorphic intratumoral vessels. According to the results, the typical pattern of hypervascularization normally seen in HCC was most frequent in moderately differentiated tumors and only partly present in well differentiated carcinomas (**Fig. 5**), whereas a significant number of well differentiated together with poorly differentiated HCC showed hypoattenuation in the arterial phase. A possible explanation for these findings may be related to the gradual changes in tumor vascularization that occur during hepatic carcinogenesis, ranging from reduced blood flow during early angiogenesis in well differentiated tumors to increased vascularization for those moderately differentiated and continuing with a decrease in blood supply as the tumor becomes less differentiated. Furthermore, the correlation between the length of time necessary for contrast washout during portal phase and differentiation grade (faster washout as tumor grading increases) allows a better tumor characterization and is a valuable tool for prognosis and a correct treatment scheme [**13**]. Other studies suggest that further optimization can be achieved by assessing tumor washout both in the portal as well as in the delayed phase because certain tumors, especially the smaller ones, present late hypoattenuation and can remain unnoticed (**Fig. 6**) [**14**,**15**].

Currently, there is a growing interest in determining the feasibility and effectiveness of PET-CT in HCC detection and also in establishing which metabolic tracer offers the most accurate results. Due to the fact that this method focuses mainly on metabolism intensity, quantified through the standardized uptake value (SUV), and less on anatomical morphology, it is commonly combined with MDCT in order to optimally pinpoint the location of areas with atypical metabolic rates [**16**]. These abnormalities are usually specific to tumorous tissue. Substances such as 18 Fluorine - Fluorodeoxyglucose (FDG) present a higher uptake in malignant cells, yet due to the normally increased glucose metabolism of hepatocytes, there are some challenges in successfully applying PET-CT as a diagnostic tool for liver carcinomas [**17**]. It has been proven that PET-CT is essential in establishing the extent of metastasis (especially lung and bone metastases) in patients already diagnosed with HCC, therefore offering a reliable long-term prognosis.

However, a recent study conducted on 138 patients with confirmed liver carcinomas revealed that, when compared to other ultrasonic or tomographic diagnostic methods, PET-CT is significantly less sensitive as a detection tool [**18**]. Numerous studies have been conducted on various techniques of enhancing PET-CT detection rate of primary HCC and secondary metastatic sites. One of them focuses on a dual-phase whole body scan. Phase one consists of a full body scan 90 minutes after the tracer is injected and is followed by phase two, a second scan is performed only in the abdominal region, in order to allow an increased ratio between the tumor and the normal parenchyma SUV and a better contrast. Although analyzed only in a limited number of studies, this approach appears to be promising, as it may improve primary HCC detection by up to 20%, and allow a more exact delimitation of tumors in relation to unaffected surrounding liver tissue [**19**,**20**].

An alternative option is the association of dual tracers such as FDG and 11C-acetate, a substance involved in lipid metabolism, which increases PET-CT sensitivity. Certain tumors that remain undetected with one tracer may become visible when using another [**21**]. Despite the increase in the sensitivity of up to approximately 80%, a major drawback is that both FDG and 11C-Acetate PET-CT reach this sensitivity only for tumors of over 5 cm, whereas small tumor detection rate (1-2 cm) is less than 40% [**22**]. 

**Magnetic Resonance Imaging (MRI)**

**Fig. 7:**
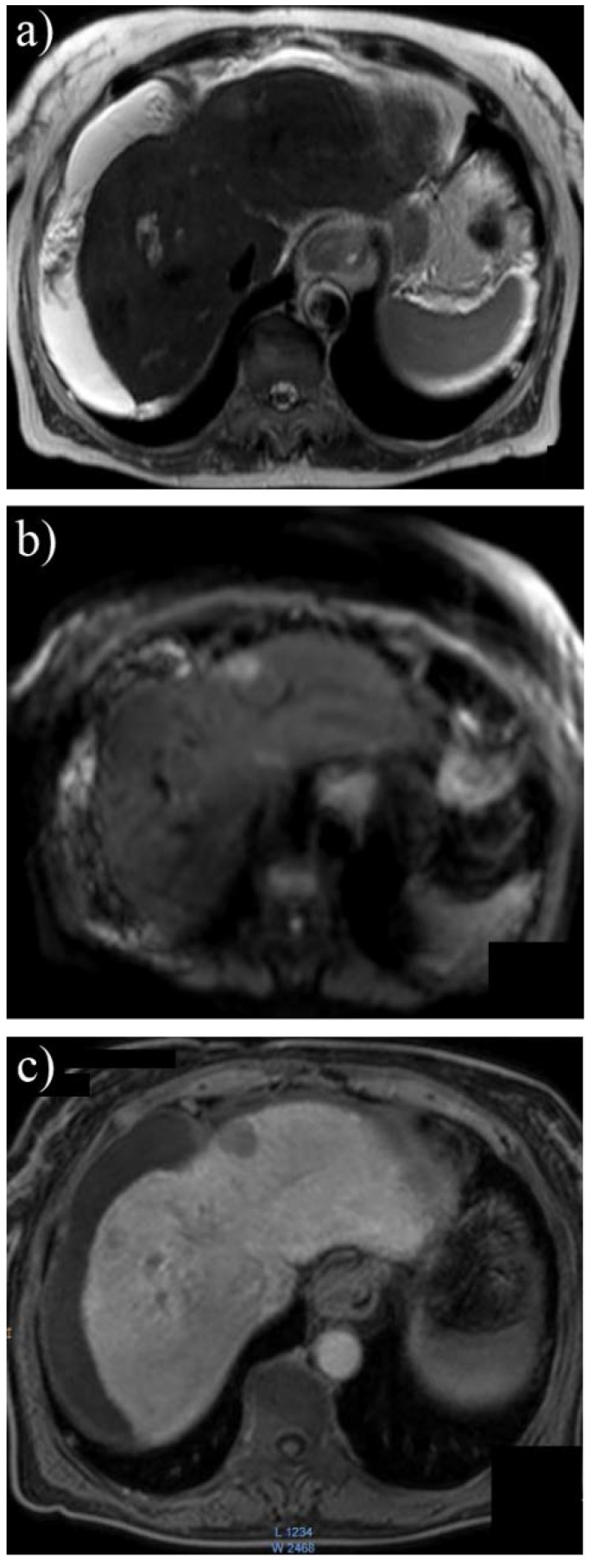
3T MRI in a patient with small HCC in segment II with high signal intensity in T2 weighted (a) and diffusion (b) sequences and decreased signal intensity at 30 minutes after contrast enhancement with hepatospecific paramagnetic gadolinium based contrast agent (c)

Diffusion weighted MRI (DW-MRI) represents another addition to the wide range of diagnostic and prognostic techniques available for patients with HCC. The functioning principle of this method is based on the diffusion capacity of water molecules, which varies depending on the density of cells in different areas of the studied tissue. As a result of the increased cellularity characteristic to tumors, liquid particles are displaced over shorter distances under the action of the magnetic field in comparison to normal cellular architecture [**23**]. DW-MRI has proven itself a more accurate tool than the conventional T1 or T2–weighted MRI [**24**]. The use of a 3T (Tesla) MRI in conjunction with subsequent Dynamic Contrast Enhanced and Diffusion are regarded as the best diagnostic tool for HCC (**Fig. 7**), although some limitations still exist for tumors under 10 mm [**25**].

**Fig. 8:**
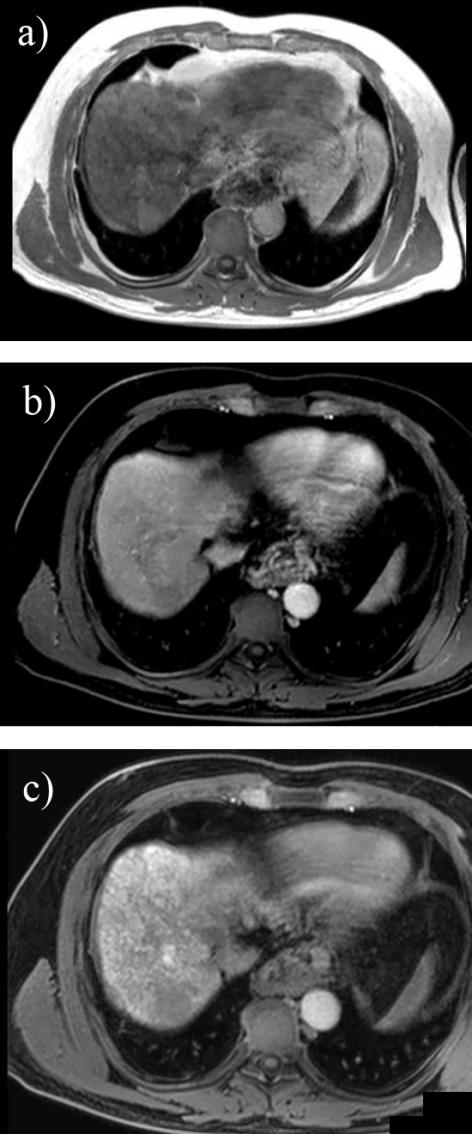
3T MRI in a cirrhotic patient using Gd-EOB-DTPA and revealing a small nodule in segment VII with high signal in axial T1 in phase (a), low signal in dynamic postcontrast (b) and at 30 minutes in hepatobilliary phase (c) suggesting the diagnosis of HCC

A 2011 study revealed that contrast-enhanced imaging such as Dynamic Contrast Enhanced MRI offers reduced sensibility for small liver tumors due to the difficulty in distinguishing venous washout phase on images less than 1 cm. Although for larger tumors, DW-MRI and Dynamic Contrast Enhanced MRI offer similar results, for lesions of this size sensibility increases from 72% to 92%, thus providing a viable option for early HCC detection [**26**]. From a different perspective, two studies that assessed 162 tumor nodules and 83 respectively showed that, although DW-MRI is more susceptible to artifacts than MDCT, its greater contrast offers comparable or even superior sensibility in liver carcinoma diagnosis. Furthermore, for small HCC (<10 mm), this novel MRI detection instrument is twice as accurate as the 64-row multidetector CT [**27**]. With the development of hepatocyte-specific magnetic resonance imaging contrast agents such as gadoxetic acid disodium (Gd-EOB-DTPA), there is an interest in combining multiple MRI techniques in order to obtain the best possible sensibility without affecting specificity, especially in hipovascular tumor (**Fig. 8**).

**Fine Needle Aspiration Liver Biopsy**

Current diagnostic guidelines of the American Association for the Study of Liver Diseases (AASLD) refer to liver biopsy only after other less invasive methods have been exhausted. According to a 2010 update, these methods now solely include imaging methods, as the use of AFP as a diagnostic criterion has been abandoned due to its reduced specificity and no other serum markers have yet been proven a viable replacement. There is a consensus that for hepatic tumors larger than 2 cm, one contrast enhanced imaging diagnostic tool, either MDCT or Dynamic Contrast Enhanced MRI, presenting a typical vascularization pattern is sufficient for diagnosis. The AASLD and European Association for the Study of the Liver guidelines have excluded CEUS from this list due to false positive results in cases with cholangiocarcinoma [**28**]. This is opposed to the Asian Pacific Association for the Study of the Liver and EFSUMB (European Federation of Societies for Ultrasound in Medicine and Biology) guidelines [**10**,**29**] which consider CEUS comparable to MDCT as previously discussed studies have shown [**30**]. For tumors between 1 and 2 cm, there are various approaches ranging from noninvasive use of one or two imaging techniques in conjunction with invasive liver biopsy, which remains the option of choice in case of uncertainty. Lesions under 1 cm remain under periodic surveillance (every 6 months) until changes in size are observed, although the Korean Liver Cancer Study Group guidelines currently consider that combining MDCT with Gadoxetic Acid Enhanced MRI is successful in detecting even infracentimetric HCCs [**31**].

As previously stated, fine needle aspiration (FNA) liver biopsy is reserved only for atypical HCC due to the inherent risks involved in any invasive procedure such as intraperitoneal or needle tract-seeding and possible bleeding. Percutaneous FNA accuracy has been reported to be up to 100%, granted that the biopsy has been taken from the suspected tissue and not from normal liver parenchyma. These errors can be avoided by performing a CT or ultrasound guided FNA. The development of endoscopic ultrasound guided FNA has largely increased the area of the liver available for biopsy and has offered a fast and relatively safe method for the histopathological diagnosis of HCC [**32**].

**Prognostic Markers**

Among the earliest discovered oncofetal proteins, AFP is currently one of the most frequently used serum tests for early stage HCC and, although groundbreaking biomarkers are being discovered, to this date, it still plays an important role in terms of HCC detection. The introduction of new serum markers in order to improve or replace AFP is encouraged by its poor specificity and sensibility coupled with the fact that recent studies have yet to provide sufficient unbiased data necessary for establishing it alone as an efficient surveillance, diagnostic and prognostic tool [**33**]. A recent study that proposed the associating AFP with human cervical cancer proto-oncogene 1 (HCCR-1) determined that the latter is actually more accurate to some extent and that a large number of subjects were positive for at least one of the two markers, which lead to a significant increase in early stage HCC diagnosis [**34**]. Furthermore, another diagnostic option for patients with AFP < 20ng/ ml is the alpha-fetoprotein isoform 3 (AFP-L3) fraction, which presents a higher specificity (80%-97%) and has proven to be useful in clinical practice [**35**]. Regarding the prognostic value of serum markers, Survivin, an apoptosis inhibitor and a promising therapeutic target, although significantly correlated with the clinical stage, portal thrombi and lymph node metastasis, has not proven to be more reliable than AFP [**36**].

Despite its modest sensitivity in preclinical tumor detection at its current cut-off values [**37**], des-gamma-carboxy-prothrombin (DCP, an abnormal prothrombin) has been suggested as a measurement for cancer progression. Tumor size, as well as vascular invasiveness and metastasis frequency, have been reported to correlate with serum DCP levels [**38**]. Moreover, increased DCP values are an indicator for the reoccurrence and low survival probability. The applicability of this marker is limited by the fact that it has a low positivity rate for small HCC with a current sensitivity of around 50%, despite the introduction of more accurate detection techniques [**39**]. A case-control study conducted on 1031 patients concluded that the sensitivity of DCP and AFP increases with HCC progression and proposed further studies regarding the use of a combination of the two markers in early diagnosis and surveillance of HCC’s response to treatment [**40**].

Numerous other biomarkers have been also studied and compared to AFP. One of these is Golgi protein 73 (GP73), a transmembrane protein, which has yielded contradictory data in several studies and thus research is required into whether it is more accurate than the traditional serum markers [**41**,**42**]. A novel study has indicated that the use of GP73 in conjunction with gamma-glutamyl transpeptidase II (GGT-II) alone did not lead to any conclusive results; however, a triple marker assay including AFP yielded promising results [**43**]. A 2007 study adopted a new approach by establishing a prognostic correlation between HCC tumor stage and aggressiveness and interleukin 18 (IL-18), which is speculated to be part of an antitumor response by adjacent healthy hepatocytes [**44**]. According to another recent study, one option would be an extensive serum and urine metabolite profiling technique, which would take into consideration various alterations of metabolic pathways determined by HCC evolution, which unfortunately may also occur in hepatitis and cirrhosis. Although a promising screening tool, this approach, as well as most of the before mentioned markers, requires further research and refining in order to be useful in clinical settings [**45**].

## Conclusions

In conclusion, the multitude of current imaging techniques available for HCC assessment allows a higher rate of detection and significantly contributes to a more accurate long-term prognosis. Contrast-enhanced ultrasonography has proven to be a cheaper, faster, simpler, non-invasive and safer method for the patient, which offers high accuracy for the characterization of focal hepatic lesions. In order to obtain a precise characterization of HCC, MRI and CT capabilities are continuously evolving and offering better results from both a morphological point of view through DW-MRI and MDCT, as well as from a functional perspective through PET-CT.

Nevertheless, there is hope that future constant research for a rapid and specific screening serum marker would lead to an early detection of small liver tumors and thus to a better chance of a successful treatment.

**Acknowledgments**

This paper was published under the frame of European Social Fund, Human Resources Development Operational Program 2007-2013, project no. POSDRU/159/1.5/136893 and UEFISCDI Partnership project VIP SYSTEM, ID: 2011–3,2-0503.

## References

[1] Marrero JA, Feng Z, Wang Y (2009). Alpha-fetoprotein, des-gamma carboxyprothrombin, and lectin-bound alpha-fetoprotein in early hepatocellular carcinoma. Gastroenterology.

[2] Ariff B, Lloyd CR, Khan S (2009). Imaging of liver cancer. World Journal of Gastroenterology: WJG.

[3] Eusebi P (2013). Diagnostic accuracy measures. Cerebrovascular Diseases.

[4] Jang HJ, Yu H, Kim TK (2009). Contrast-enhanced ultrasound in the detection and characterization of liver tumors. Cancer imaging: the official publication of the International Cancer Imaging Society.

[5] Danila M, Sporea I, Sirli R (2010). The role of contrast enhanced ultrasound (CEUS) in the assessment of liver nodules in patients with cirrhosis. Medical Ultrasonography.

[6] Sporea I, Badea R, Popescu A (2014). Contrast-enhanced ultrasound (CEUS) for the evaluation of focal liver lesions - a prospective multicenter study of its usefulness in clinical practice. Ultraschall in der Medizin.

[7] Sorrentino P, D'Angelo S, Tarantino L (2009). Contrast-enhanced sonography versus biopsy for the differential diagnosis of thrombosis in hepatocellular carcinoma patients. World Journal of Gastroenterology: WJG.

[8] Xu JF, Liu HY, Shi Y (2011). Evaluation of hepatocellular carcinoma by contrast-enhanced sonography: correlation with pathologic differentiation. Journal of ultrasound in medicine: official journal of the American Institute of Ultrasound in Medicine.

[9] Seitz K, Strobel D, Bernatik T (2009). Contrast-Enhanced Ultrasound (CEUS) for the characterization of focal liver lesions - prospective comparison in clinical practice: CEUS vs. CT (DEGUM multicenter trial). Parts of this manuscript were presented at the Ultrasound Dreilandertreffen 2008, Davos. Ultraschall in der Medizin.

[10] Claudon M, Dietrich CF, Choi BI (2013). Guidelines and good clinical practice recommendations for Contrast Enhanced Ultrasound (CEUS) in the liver - update 2012: A WFUMB-EFSUMB initiative in cooperation with representatives of AFSUMB, AIUM, ASUM, FLAUS and ICUS. Ultrasound in Medicine & Biology.

[11] Sandulescu L, Saftoiu A, Dumitrescu D (2009). The role of real-time contrast-enhanced and real-time virtual sonography in the assessment of malignant liver lesions. Journal of gastrointestinal and liver diseases: JGLD.

[12] Kopp AF, Heuschmid M, Claussen CD (2002). Multidetector helical CT of the liver for tumor detection and characterization. European Radiology.

[13] Lee JH, Lee JM, Kim SJ (2012). Enhancement patterns of hepatocellular carcinomas on multiphasic multidetector row CT: comparison with pathological differentiation. The British Journal of Radiology.

[14] Furlan A, Marin D, Vanzulli A (2011). Hepatocellular carcinoma in cirrhotic patients at multidetector CT: hepatic venous phase versus delayed phase for the detection of tumour washout. The British Journal of Radiology.

[15] Iannaccone R, Laghi A, Catalano C (2005). Hepatocellular carcinoma: role of unenhanced and delayed phase multi-detector row helical CT in patients with cirrhosis. Radiology.

[16] Sun L, Wu H, Guan YS (2007). Positron emission tomography/computer tomography: challenge to conventional imaging modalities in evaluating primary and metastatic liver malignancies. World Journal of Gastroenterology: WJG.

[17] Lan BY, Kwee SA, Wong LL (2012). Positron emission tomography in hepatobiliary and pancreatic malignancies: a review. American Journal of Surgery.

[18] Lee JE, Jang JY, Jeong SW (2012). Diagnostic value for extrahepatic metastases of hepatocellular carcinoma in positron emission tomography/ computed tomography scan. World Journal of Gastroenterology: WJG.

[19] Lin WY, Tsai SC, Hung GU (2005). Value of delayed 18F-FDG-PET imaging in the detection of hepatocellular carcinoma. Nuclear Medicine Communications.

[20] Kuker RA, Mesoloras G, Gulec SA (2007). Optimization of FDG-PET/ CT imaging protocol for evaluation of patients with primary and metastatic liver disease. International Seminars in Surgical Oncology: ISSO.

[21] Larsson P, Arvidsson D, Bjornstedt M (2012). Adding 11C-acetate to 18F-FDG at PET Examination Has an Incremental Value in the Diagnosis of Hepatocellular Carcinoma. Molecular Imaging and Radionuclide Therapy.

[22] Park JW, Kim JH, Kim SK (2008). A prospective evaluation of 18F-FDG and 11C-acetate PET/ CT for detection of primary and metastatic hepatocellular carcinoma. Journal of nuclear medicine: official publication. Society of Nuclear Medicine.

[23] Kele PG, van der Jagt EJ (2010). Diffusion weighted imaging in the liver. World Journal of Gastroenterology: WJG.

[24] Yang DM, Jahng GH, Kim HC (2011). The detection and discrimination of malignant and benign focal hepatic lesions: T2 weighted vs diffusion-weighted MRI. The British Journal of Radiology.

[25] Kim DJ, Yu JS, Kim JH (2012). Small hypervascular hepatocellular carcinomas: value of diffusion-weighted imaging compared with “washout” appearance on dynamic MRI. The British Journal of Radiology.

[26] Kim YK, Kim CS, Han YM, Yu HC, Choi D (2011). Detection of small hepatocellular carcinoma: intraindividual comparison of gadoxetic acid-enhanced MRI at 3.0 and 1.5 T. Invest Radiol.

[27] Pitton MB, Kloeckner R, Herber S (2009). MRI versus 64-row MDCT for diagnosis of hepatocellular carcinoma. World Journal of Gastroenterology: WJG.

[28] (2012). 28. European Association For The Study Of The L, European Organization For R, Treatment Of C. EASL-EORTC clinical practice guidelines: management of hepatocellular carcinoma. Journal of Hepatology.

[29] Omata M, Lesmana LA, Tateishi R (2010). Asian Pacific Association for the Study of the Liver consensus recommendations on hepatocellular carcinoma. Hepatology International.

[30] Bruix J, Sherman M (2011). American Association for the Study of Liver D. Management of hepatocellular carcinoma: an update. Hepatology.

[31] Song do S, Bae SH (2012). Changes of guidelines diagnosing hepatocellular carcinoma during the last ten-year period. Clinical and Molecular Hepatology.

[32] Wee A (2011). Fine-needle aspiration biopsy of hepatocellular carcinoma and related hepatocellular nodular lesions in cirrhosis: controversies, challenges, and expectations. Pathology Research International.

[33] Gorog D, Regoly-Merei J, Paku S (2005). Alpha-fetoprotein expression is a potential prognostic marker in hepatocellular carcinoma. World Journal of Gastroenterology: WJG.

[34] Zhang G, Ha SA, Kim HK (2012). Combined analysis of AFP and HCCR-1 as an useful serological marker for small hepatocellular carcinoma: a prospective cohort study. Disease Markers.

[35] Choi JY, Jung SW, Kim HY (2013). Diagnostic value of AFP-L3 and PIVKA-II in hepatocellular carcinoma according to total-AFP. World Journal of Gastroenterology: WJG.

[36] El-Attar HA, Kandil MH, El-Kerm YM (2010). Comparison of serum survivin and alpha fetoprotein in Egyptian patients with hepatocellular carcinoma associated with hepatitis C viral infection. Asian Pacific Journal of Cancer Prevention: APJCP.

[37] Mittal A, Gupta SP, Sathian B (2012). Des-gamma-carboxyprothrombin for early identification and prognosis of hepatocellular carcinoma--a case control study from western Nepal. Asian Pacific Journal of Cancer Prevention: APJCP.

[38] Nagaoka S, Yatsuhashi H, Hamada TJH (2003). The des-gamma-carboxy prothrombin index is a new prognostic indicator for hepatocellular carcinoma. Cancer.

[39] Hakamada K, Kimura N, Miura T (2008). Des-gamma-carboxy prothrombin as an important prognostic indicator in patients with small hepatocellular carcinoma. World Journal of Gastroenterology: WJG.

[40] Lok AS, Sterling RK, Everhart JE (2010). Des-gamma-carboxy prothrombin and alpha-fetoprotein as biomarkers for the early detection of hepatocellular carcinoma. Gastroenterology.

[41] Zhou Y, Yin X, Ying J (2012). Golgi protein 73 versus alpha-fetoprotein as a biomarker for hepatocellular carcinoma: a diagnostic meta-analysis. BMC Cancer.

[42] Ba MC, Long H, Tang YQ (2012). GP73 expression and its significance in the diagnosis of hepatocellular carcinoma: a review. International Journal of Clinical and Experimental Pathology.

[43] Hou SC, Xiao MB, Ni RZ (2013). Serum GP73 is complementary to AFP and GGT-II for the diagnosis of hepatocellular carcinoma. Oncology Letters.

[44] Tangkijvanich P, Thong-Ngam D, Mahachai V (2007). Role of serum interleukin-18 as a prognostic factor in patients with hepatocellular carcinoma. World Journal of Gastroenterology: WJG.

[45] Chen T, Xie G, Wang X (2011). Serum and urine metabolite profiling reveals potential biomarkers of human hepatocellular carcinoma. Molecular & Cellular Proteomics: MCP.

